# The underdeveloped population dimensions of inoculation theory in public health

**DOI:** 10.1093/eurpub/ckag195

**Published:** 2026-10-30

**Authors:** Josh Compton

**Affiliations:** Speech at Dartmouth, Dartmouth College, Hanover, NH, United States

Inoculation theory—a longstanding theory of resistance to influence [1]—explains how exposure to weakened challenges can prepare people to resist stronger challenges encountered later, and it has had health at its centre from the start. The theory is named after a health practice—medical inoculation—and its earliest applications included health beliefs, such as the importance of annual medical exams [1]. Over its 60-plus-year history, health has remained one of the most common areas for applied inoculation, with smoking and alcohol prevention especially prominent in the 1980s, 1990s, and early 2000s, then later expanding into areas such as vaccination, exercise, and safer-sex promotion in the 2010s through today [2].

Much of this work is at the individual-behavioural level. This important work should continue. But other aspects of health, including public health, need attention, too. The groundwork is there. Inoculation theory has been tested as a policy messaging strategy around health-policy issues in the USA, including tobacco regulation, sugar-sweetened beverage taxes, and prescription painkiller addiction policy [3], and other efforts have explored inoculation-informed interventions at scale, such as gamified inoculation to mitigate misinformation about health-related issues [4].

Despite these overlaps, inoculation theory’s population-level dimensions remain comparatively underdeveloped in public health. Although recent public-health discussions increasingly invoke inoculation and prebunking as strategies against misinformation [5], the larger theoretical framework from which these approaches emerged remains comparatively underexplored.

Certainly, communication across networks isn’t exclusive to inoculation theory. Other frameworks also explain how messages and influence move through social networks [6]. But inoculation raises a more specific question: what happens when resistance to future influence, created through pre-emptive exposure to weakened challenges, moves through these social processes?

More engagement with public health theory and research would strengthen inoculation theory and its engagement with public health practice. For example, such work could build on evidence of inoculation’s efficacy while drawing more directly on public health communication research on interactive, transactional, and two-way message circulation [7].

This population-level turn is consistent with inoculation theory’s basic premise—exposure to weakened challenges builds resistance to stronger ones—while directing more explicit attention to how resistance operates at the societal level alongside the well-studied individual-level effects. Public health may be most useful to inoculation theory not simply by adding new issues and topics, but by continuing to shift the scale of inquiry.

Postinoculation talk offers one place to begin. Postinoculation talk refers to the communication that occurs after an inoculation-based intervention. Existing research shows that postinoculation talk can strengthen resistance to influence [8]. Yet much of what we know of postinoculation talk still ends at the individual level: how many people one talked to, how many conversations were had, and the effects on the individual’s position. That is largely where the empirical record stops. Postinoculation talk research now needs to follow the message farther.

That broader view brings longstanding but underexamined questions into focus [9]. Can an inoculation intervention affect people who never encountered the original intervention? How does resistance to influence change as it moves through interpersonal networks, families, organizations, and communities? Under what conditions might those resistance effects accumulate, weaken, or change along the way? These are questions about resistance to influence, but they are also of clear public health relevance because they concern how communication interventions circulate beyond the individuals who directly experience them.

It’s a critical difference. Inoculation interventions can be carefully controlled at initial exposure, because researchers can balance an advocated position with weakened counterarguments and refutations. That control diminishes once inoculated individuals become sources themselves. We know relatively little about what happens as resistance moves from person to person, much less from community to community, although initial modelling work has examined inoculation effects in large rational agent networks [10].

Inoculation theory’s long history has set us up for a particularly exciting opportunity—to situate inoculation more explicitly within public health theory and practice. This doesn’t mean abandoning individual-level questions, which remain fundamental to the theory and its continuing evolution. But it could mean extending these questions from how individuals resist to how resistance builds and changes through communication among people, to how communities and institutions can reduce shared vulnerability to recurring harmful challenges. Public health offers an opportunity for inoculation theory to keep following the message after the intervention ends.

Finally, this population-level turn may also guide the practice of effective public health communication, especially as a way to mitigate misinformation [4]. Magid *et al.* [5] call for strengthening communication capacities within the public health workforce so that professionals are better prepared to counter misinformation. Inoculation offers one framework. Pre-emptively raising and refuting misinformation, including common misinformation strategies [4], can help prepare public health professionals and the communities they serve before stronger challenges are encountered. Developing these population-level dimensions could benefit both inoculation theory and public health practice.

## Data Availability

Not applicable. No datasets were generated or analysed for this article.
